# Laser micropatterned dermal templates support early rete ridge formation and basement membrane deposition when used with cultured epithelial autografts

**DOI:** 10.1016/j.burns.2025.107613

**Published:** 2025-07-12

**Authors:** Britani N. Blackstone, Molly E. Baumann, Summer C. Gallentine, Dorothy M. Supp, J. Kevin Bailey, Heather M. Powell

**Affiliations:** a Department of Materials Science and Engineering, The Ohio State University, 3024 Fontana Labs, 140 W 19th Ave, Columbus, OH 43210, USA; b Department of Biomedical Engineering, The Ohio State University, 2124 Fontana Labs, 140 W 19th Ave, Columbus, OH 43210, USA; c Shriners Children’s Ohio, 1 Children’s Plaza 2 West, Dayton, OH 45404, USA; d University of Cincinnati College of Medicine, Department of Surgery, 231 Albert Sabin Way, P.O. Box 670558, Cincinnati, OH 45267, USA; e Center for Stem Cell & Organoid Medicine (CuSTOM), Cincinnati Children’s Hospital Medical Center, 3333 Burnet Ave #7015, Cincinnati, OH 45229, USA; f Department of Surgery, Wake Forest University School of Medicine, 475 Vine Street, Winston-Salem, NC 27101, USA

**Keywords:** Scaffold, Laser ablation, Rete ridge, Engineered skin, Basement membrane, Biomechanics

## Abstract

The success of cultured epithelial autografts (CEAs) in treating large surface area burns is limited by their fragility and poor adhesion to the wound bed. Rete ridges, interdigitations at the dermal-epidermal junction, are stem cell niches critical to dermal-epidermal adhesion and distribution of mechanical forces within the skin, but are slow to develop in CEA-treated burn wounds. A porcine burn-excise-autograft model was used to investigate the efficacy of an allogeneic dermal template (DT), seeded with fibroblasts and laser micropatterned to have dermal papillae-like topography, in improving outcomes with CEA. Autologous CEAs were applied to full-thickness wounds alone or with DTs that were flat (CEA+Flat) or micropatterned (CEA+Ridged). The use of a DT improved graft handleability and integration at the wound margins, and restored epidermal barrier function two weeks faster than CEAs alone. In CEA+Ridged grafts, increases in keratinocyte proliferation and basement membrane deposition were observed at 2 weeks post-grafting. Rete ridges were only present in CEA+Ridged grafts at week 2, and developed less frequently and shallower in CEA and CEA+Flat grafts over the course of the study. These results suggest that laser micropatterned DTs are suitable for co-transplantation with CEAs and can significantly improve graft adhesion and development.

## 1. Introduction

Patients with large surface area burns lack sufficient donor tissue for routine split-thickness skin autografting and, subsequently, are more likely to suffer from complications such as infection and scarring. Isolation and expansion of autologous cells from skin biopsies *in vitro* supports alternative treatment options such as cultured epithelial autografts (CEA), which can increase the treatable area up to 300 times the initial donor biopsy size [1]. However, because CEAs lack a dermal component, their poor biomechanics and slow basement membrane formation make them extremely susceptible to damage during handling and grafting, as well as post-grafting where they can easily shear or blister [2–6]. Outcomes for CEAs are highly variable, and graft take rates between 0 % and 100 % have been reported [7–11].

Improvements in outcomes have been observed when CEAs were used in conjunction with a dermal matrix or meshed and expanded split-thickness skin grafts (STSG) [12–14]. When used with an acellular dermal matrix (Integra^®^ or Terudermis^®^), pairing CEAs with a 1:6 meshed skin graft reduced the amount of donor tissue needed without sacrificing scar quality when compared with a 1:3 meshed skin graft, though an additional 13 days were needed for healing [15]. Heard et al. found that the addition of biodegradable temporizing matrix (BTM) to the wound bed prior to CEA placement resulted in an average wound closure of 95 % at day 135 ± 35 [16]. However, five of the ten patients required removal and/or replacement of the BTM, four of which were due to infection, and graft take was highly variable when CEA was applied without a STSG [16]. Grafting CEAs onto an acellular collagen substrate (Integra^®^) supported successful engraftment to full thickness burn wounds; however, basement membrane proteins were slow to develop and rete ridges—interdigitations of epidermis and dermis that improve the mechanical properties of skin—were not observed at day 294 post-grafting [5]. Alloderm^®^, a decellularized human dermal matrix that preserves the dermal papilla architecture of the tissue, has also been shown to improve outcomes for CEAs long-term [17,18], decreasing joint contracture, scar thickness and erythema [19]. Limitations for this type of product are related to tissue availability and heterogeneity, as well as the possibility of unwanted side effects of the decellularization process, such as tissue damage or disease transmission.

Prior histological analyses of CEAs show slow basement protein deposition and late formation of dermal papillae/rete ridges, if observed [5]. Dermal papillae and epidermal rete ridges play important roles in skin homeostasis and healing, acting as niches for wound healing and homeostasis associated epidermal stem cells [20–22], and enhancing dermal-epidermal adhesion and crosstalk [6]. This architecture has largely been ignored in the development of dermal and skin substitutes, though recent tissue engineering strategies have been exploring the best ways to develop these structures [23–32]. A fractional carbon dioxide (FXCO_2_) laser has previously been used to micropattern human fibroblast-seeded dermal templates used in the development of engineered skin with rete ridges [29,33] and as an artificial dermal carrier for CEAs [30]. These *in vitro* and murine studies suggested that the laser micropatterning process does not increase inflammation within the dermal template or post-grafting [33]. Further, the micropatterned templates facilitated rete ridge formation within two weeks of grafting in conjunction with CEAs, in contrast to CEAs grafted with flat templates, which did not form rete ridges by four weeks [30]. In that study, increased keratinocyte proliferation, epidermal thickness, and epidermal stemness in the CEAs grafted with micropatterned DTs [30] were observed.

An established porcine burn model [34,35] was used in this study to test the hypothesis that fibroblast-seeded laser micropatterned dermal templates can improve the outcomes for CEAs grafted onto excised, full-thickness burn wounds, by providing a larger surface area for CEAs to attach and facilitating fibroblast-keratinocyte communication. Autologous porcine fibroblasts and keratinocytes were isolated from split-thickness skin harvested from each pig. Fibroblasts were expanded, pooled and seeded onto freeze-dried collagen scaffolds to form allogeneic porcine dermal templates (DTs), and half of the templates were laser micropatterned to achieve a ridged architecture prior to grafting. Autologous CEAs were grafted onto wound sites in three treatment groups: CEA alone, CEA combined with a flat DT, and CEA combined with a ridged DT. Graft contraction, erythema, pigmentation, vascularization, biomechanics and basement membrane formation were assessed for nine weeks.

## 2. Materials and methods

### 2.1. Porcine tissue collection

**Fig. 1.**
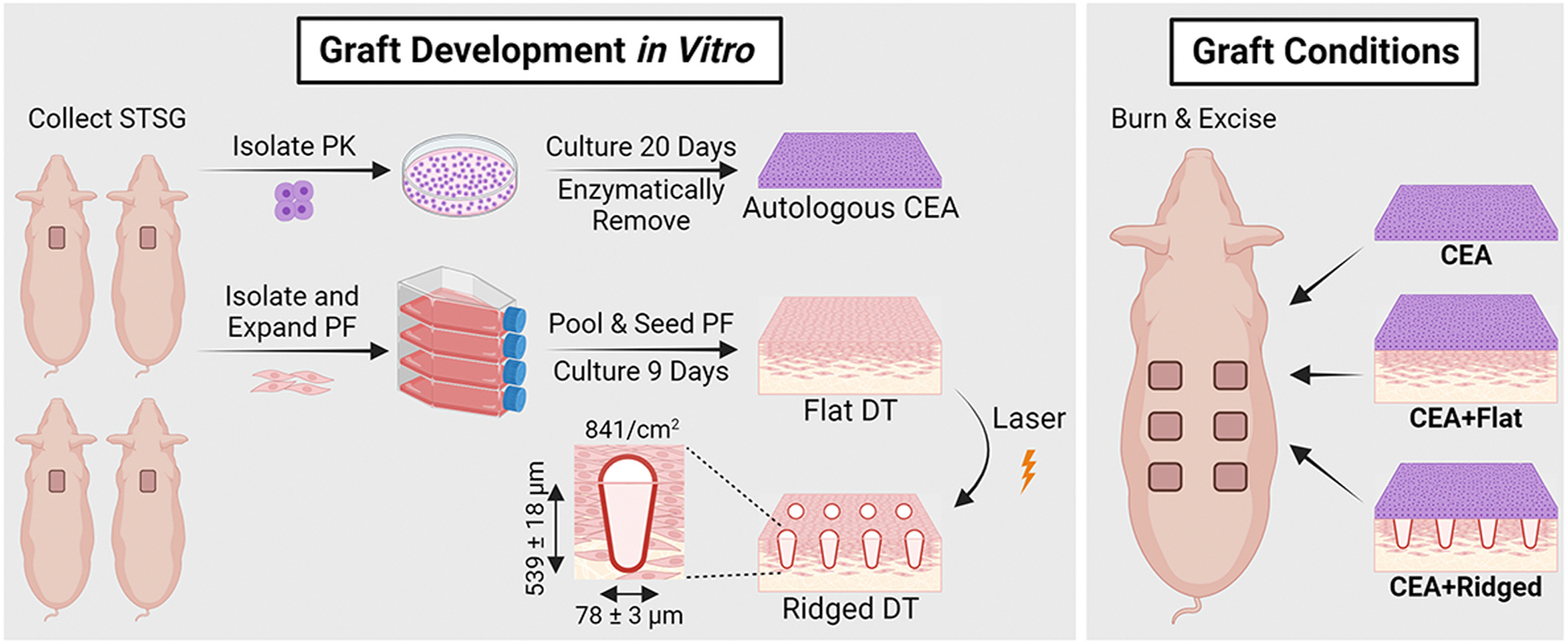
Method for developing CEA, CEA+Flat and CEA+Ridged grafts from split thickness skin grafts. CEAs, Flat dermal templates and Ridged dermal templates were cultured and fabricated *in vitro* and applied to excised burn sites as CEA grafts alone (CEA, control) or in combination with Flat (CEA+Flat) or Ridged (CEA+Ridged) DTs. Created in BioRender. Powell, H. (2025) BioRender.com/y44p476.

The Ohio State University Institutional Laboratory Animal Care and Use Committee (IACUC) approved all protocols for experiments and data collection. Tissue was collected from each of four female red duroc pigs (Isler Genetics, Inc., Prospect, Ohio) for cell isolation and subsequent creation of cultured grafts. Following anesthetization, the dorsal midline of the trunk was shaved and sterilized with two alternating scrubs of 2 % chlorhexidine and 70 % alcohol. Skin samples were then harvested with a ZimmerAir Dermatome at 0.010” thickness and stored in sterile HEPES buffered saline (HBS) on ice (Fig. 1). Restore Contact Layer FLEX^™^ (Hollister Wound Care, Libertyville, IL) was used as an antibacterial dressing on the donor sites. Vetrap^™^ (3 M, St. Paul, MN) was wrapped around the trunks of the pigs to hold the Restore in place and protect the wound sites, and Elastikon^®^ (Johnson & Johnson, New Brunswick, NJ) was used to secure the Vetrap^™^. An intramuscular injection of buprenorphine was administered to each pig for pain management, as well as a fentanyl patch (NOVAPLUS path, Watson Pharmaceuticals Inc., Pasippany, NJ, 100 μg) placed in the ear pinna for three days post-wounding. Dressings were removed 7 days after tissue collection. Animals were housed individually and received standard chow *ad libitum* while on study, with overnight fasts prior to each procedure.

### 2.2. Cell isolation

Porcine fibroblasts (PF) and keratinocytes (PK) were isolated [36,37] and cultured [38] from each piece of collected tissue according to previously described methods for human skin cells. Briefly, the skin was disinfected in a 5 % chloroxylenol (Dettol, Slough, UK), rinsed for 20 s and rinsed twice with HBS. The tissue was then cut into 2 mm-wide strips and exposed to an overnight soak of Dispase II (2.4 U/mL in HBS, ThermoFisher Scientific, Waltham, MA) at 4°C. The epidermis and dermis of each strip were then mechanically separated. Dermal strips were minced and incubated in collagenase type I (625 U/mL, ThermoFisher Scientific) for 60 min. Dermal tissue was rinsed, plated in flasks, and subcultured for PF expansion. PKs were isolated from the epidermis with trypsin-EDTA, counted and plated onto 100-mm dishes at 1 × 10^4^ cells/cm^2^. PK and PF from each animal were cultured separately with PF pooled prior to dermal template fabrication. Culture medium [38] was exchanged for both cell types at least every 48 h.

### 2.3. Formation of porcine dermal templates and cultured epithelial autografts

Freeze dried collagen scaffolds were used to construct the dermal templates. Scaffolds were fabricated as previously described [39,40] by lyophilizing a frozen solution of 0.6 % wt./vol. bovine collagen powder (SEMED F, Kensey Nash, Exton, Pa) in 0.5 M acetic acid. Scaffolds (3 cm × 3 cm) were physically cross-linked via dehydrothermal treatment for 24 hr at 140°C, disinfected in 70 % ethanol for 24 hr, rinsed with HBS five times and cell culture medium twice. Nine days prior to grafting, isolated fibroblasts were collected, pooled in equal numbers from each animal and seeded onto the scaffolds at 5 × 10^5^ cells/cm^2^. Twelve hours prior to grafting, an Ultrapulse^®^ fractional carbon dioxide laser (FXCO_2_, Lumenis, Inc., San Jose, CA) was used to create a micropattern on the surface of half of the dermal templates, utilizing the DeepFX^™^ handset (5 mJ, 25 % density, 300 Hz) [41].

Cultured epithelial autografts (CEAs) were developed by culturing keratinocytes from each individual pig in 100 mm dishes for 20 days with daily medium changes (Fig. 1). The morning of the grafting procedure, CEAs were detached from the dishes by incubation in 1.2 U/mL Dispase II (ThermoFisher Scientific) for 5 min followed by 2 rinses in HBS and subsequent use of cell scrapers to completely release the CEA from the dish. After CEAs were released they were cut into 3 × 3 cm squares. Three graft types were then developed: CEA, CEA plus a non-lasered dermal template (CEA+Flat), and CEA plus a laser-micropatterned dermal template (CEA+Ridged). Prior to grafting, CEAs alone were placed onto a non-adherent dressing N-terface^®^ (Winfield Laboratories, Inc., Richardson, TX) to facilitate transfer to the wound bed. For composite grafts (i.e., CEA + dermal template), DT (3 ×3 cm) was placed into the dish with the CEA, submerged to the bottom of the dish and the CEA floated above the DT. The DT was then raised from the medium capturing the CEA on top. N-terface was then placed on top of the composite graft; no adhesives or tack sutures were utilized to hold the CEAs onto the DTs.

### 2.4. Injury model and grafting

An established porcine burn model [34,42]was used to create wound sites for the experimental grafts. Pigs were anesthetized and their trunks were shaved and disinfected as described in *Porcine Tissue Collection*. A full-thickness wound was administered by heating a 1 × 1 in. stainless-steel stylus to 200 ± 6°C and applying it with three pounds of pressure to the dorsal skin for 25 s (n = 6/pig, 3/side). Each wound was excised to the burn margin, down to viable tissue, and treated with one of three graft conditions (CEA, CEA+Flat or CEA+Ridged). Treatments were randomized for each pig, with 2 replicates per pig, and distributed as evenly as possible among the locations (anterior, middle, posterior) as pig growth and wound contraction can vary based on location. Grafts were dressed and protected with a layer of non-adherent and a layer of gauze coated with antibiotic ointment. Bolsters for the grafts consisted of Restore Contact Layer FLEX^™^ and saline-soaked surgical sponges (Hydrasorb, Carwild Corp., New London, CT), and were secured via stapled spandex. A shell consisting of Vetrap^™^, surgical sponges, and a fiberglass cast (Scotchcast^™^, 3 M), and secured by Vetrap^™^ and Elastikon^®^, was used to protect the wound sites. A fentanyl patch and IM buprenorphine were administered as described in *Porcine Tissue Collection*.

### 2.5. Data collection

On days 4, 7, 11, 14, 18, 21, 28, 35, 47 and 63, dressings were removed, grafts were allowed to air out for at least 20 min, data analysis was performed and the sites redressed. Measurement techniques that required contact with the grafts were delayed so as not to disturb the CEAs with the probes. TEWL was delayed to day 14 post-grafting as it required only light contact with the skin’s surface; however, Mexameter measurements were delayed until day 21 as it required more pressure to acquire the measurement. Mechanical measurements were collected at days 35 and later as this required the wounds be closed. Control sites for measurements of normal pig skin were chosen at the shoulder and at the dorsal midline, in between grafts. Biopsies were collected from grafts and frozen in OCT compound on days 7, 14, 28 and 63 for histological evaluation. On day 63, after completion of *in vivo* data collection, all animals were euthanized.

### 2.6. Graft area

Photographs of the grafts were taken immediately after grafting (day 0) and at every time point, with a scale bar and a color palette in the field of view (Nikon D2700, Tokyo, Japan). Computer planimetry (ImageJ, https://imagej.nih.gov/ij/) was used to measure the graft area from the images. Values for grafts from day 7–63 were then calculated as percentages of their initial graft areas, at day 0, and reported as a normalized average ± standard deviation (SD).

### 2.7. Transepidermal water loss

At days 14, 21, 28, 35, 47 and 63 post-grafting, measurements of transepidermal water loss (TEWL) were taken 20 min after dressing removal and equilibration to ambient conditions. A Tewameter^®^ TM 300 probe (Courage + Khazaka Electronic GmbH, Köln, Germany) was used to measure TEWL on a representative area of each graft and of normal pig skin. These values are reported as average TEWL ± SD.

### 2.8. Graft pigmentation and erythema

A Mexameter^®^ (Courage + Khazaka Electronic GmbH) was used to quantify graft color for pigmentation and erythema at days 21, 28, 35, 47 and 63 post-grafting. Measurements were collected in triplicate for each graft and each normal skin site. At each time point, pigmentation and erythema values of grafts were normalized to those of normal skin for each pig and are reported as average ± SD.

### 2.9. In Vivo biomechanics

At days 35, 47 and 63, a Biomechanical Tissue Characterization system (BTC-2000^™^ Surgical Research Laboratory, Inc., Nashville, TN) was used to measure graft elasticity, laxity and stiffness as previously described [43]. Briefly, an optical laser measured skin displacement while negative pressure was applied at 10 mmHg/sec up to 150 mmHg, held for 5 sec, and released for 3 sec. A representative area from each graft and normal skin site was measured and values are reported as average ± SD.

### 2.10. Histological staining and immunostaining

Cryosections with 7 μm thickness were cut from the frozen OCT-embedded biopsies. For ridged grafts, at least 120 serial sections per sample were collected, fixed with acetone and visualized with light microscopy prior to staining to ensure representative sections were selected. To assess general tissue morphology, sections were stained with hematoxylin and eosin (H&E) and representative images are reported. Immunohistochemistry was utilized to evaluate basement membrane formation, keratinocyte proliferation and blood vessel formation. Antibodies included rabbit anti-collagen VI (Abcam, Cambridge, MA), rabbit anti-collagen VII (Abcam), rabbit anti-Von Willebrand Factor (VWF, Abcam), mouse anti-alpha smooth muscle actin (α-SMA, Invitrogen, Waltham, MA), anti-rabbit Ki67 (Invitrogen) and anti-pan cytokeratin AlexaFluor^®^ 488 conjugate (Invitrogen). AlexaFluor^®^ labeled secondary antibodies (ThermoFisher Scientific) were used to detect primary antibodies and nuclei were counter-stained with DAPI (ThermoFisher Scientific).

Immunostained samples were imaged using confocal microscopy (Olympus FV1000 Filter and Leica Stellaris). The entire epidermis of each section stained with collagen IV and collagen VII was imaged at 10x, and a representative field of view is reported. For VWF and α-SMA stained sections, at least 4 samples per graft condition and time point were stained and an entire cross-section was imaged at 2x. VWF presence in the dermis was quantified via ImageJ by normalizing the number of VWF-positive pixels to the number of pixels in selected dermal area, with section edges excluded. Representative images and the average percentage of VWF-positive pixels in the dermis ± SD are reported. For Ki67 and pan-cytokeratin stained sections, 4 samples per graft condition and time point were stained and non-overlapping images spanning at least 2 mm of tissue were captured at 10x. The number of Ki67-positive nuclei in cells that were positive for pan-cytokeratin were counted and normalized to the epidermal length calculated via ImageJ. Representative images and the average number of Ki67-positive nuclei per mm length of epidermis ± SD are reported.

### 2.11. Statistical analyses

Statistical analysis was performed using SigmaPlot v15 (Systat Software Inc., San Jose, CA). Differences within each treatment condition with time and among treatment conditions at the same time point were assessed using One-Way Analysis of Variance (ANOVA) or a One-Way ANOVA on Ranks, when data did not follow a normal distribution, both with a *post hoc* test of Tukey. Statistical significance was established with a p value < 0.05.

## 3. Results

### 3.1. CEA and CEA+DT composite formation

**Fig. 2.**
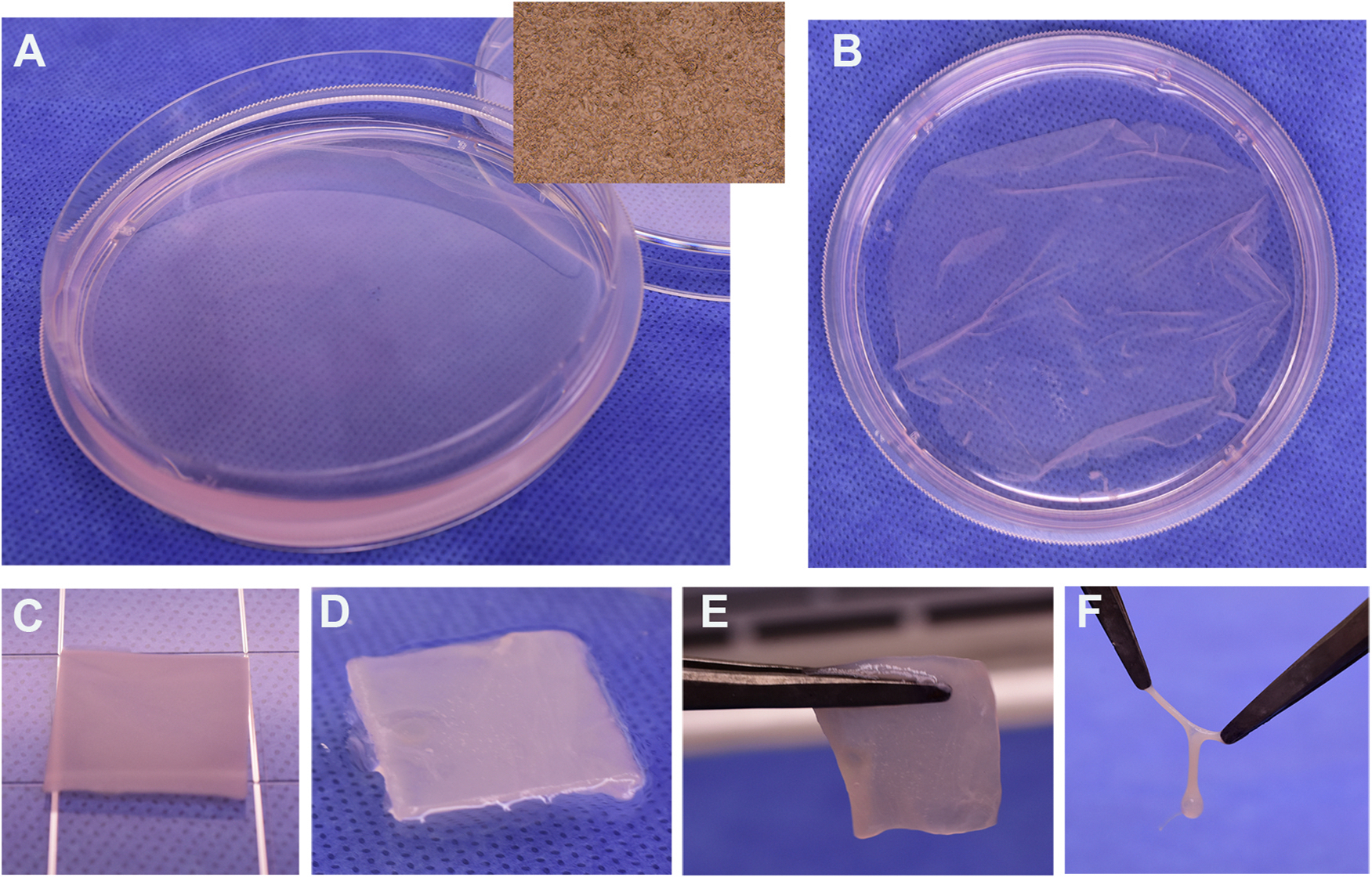
A) Photograph of the CEA at culture day 20 with polarized light microscope images of the keratinocytes within the CEA (inset). Photographs of the CEA after release (B), and the dermal template alone (C) and with the CEA on top (D). E) The CEA+DT can be manipulated with forceps; however, the CEA folds when moved (F).

All porcine keratinocytes formed CEAs that were 3–6 cell layers thick and completely covered the bottom of the 100 mm dish (Fig. 2A). Once released from the well, the CEAs became slightly smaller than the dish but remained contiguous (Fig. 2B). CEAs were placed on top of the DTs to create composite grafts that could be handled in the absence of the N-terface dressing; however, CEAs could not be handled or transferred to the wound bed alone (Fig. 2. C–F).

### 3.2. Graft assessment and morphology

**Fig. 3.**
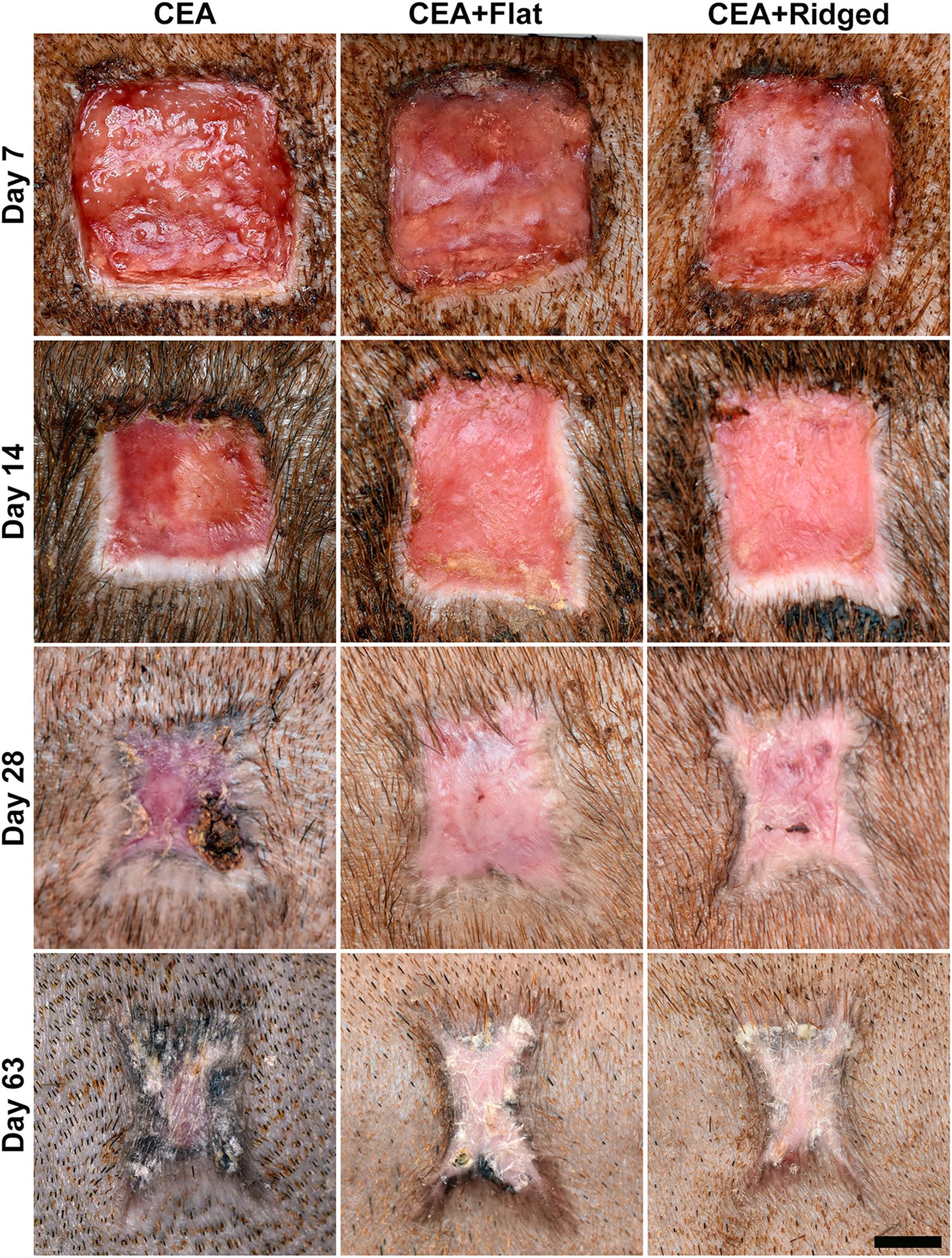
Representative photographs of CEA, CEA+Flat and CEA+Ridged grafts at 7, 14, 28, and 63 days post-grafting. All grafts contracted over time. Little pigmentation was observed in the center of the grafts, with increased pigmentation at grafts borders, particularly in the CEA group. Erythema appeared to be increased in CEA group up to day 28. Scale bar = 1 cm.

**Fig. 4.**
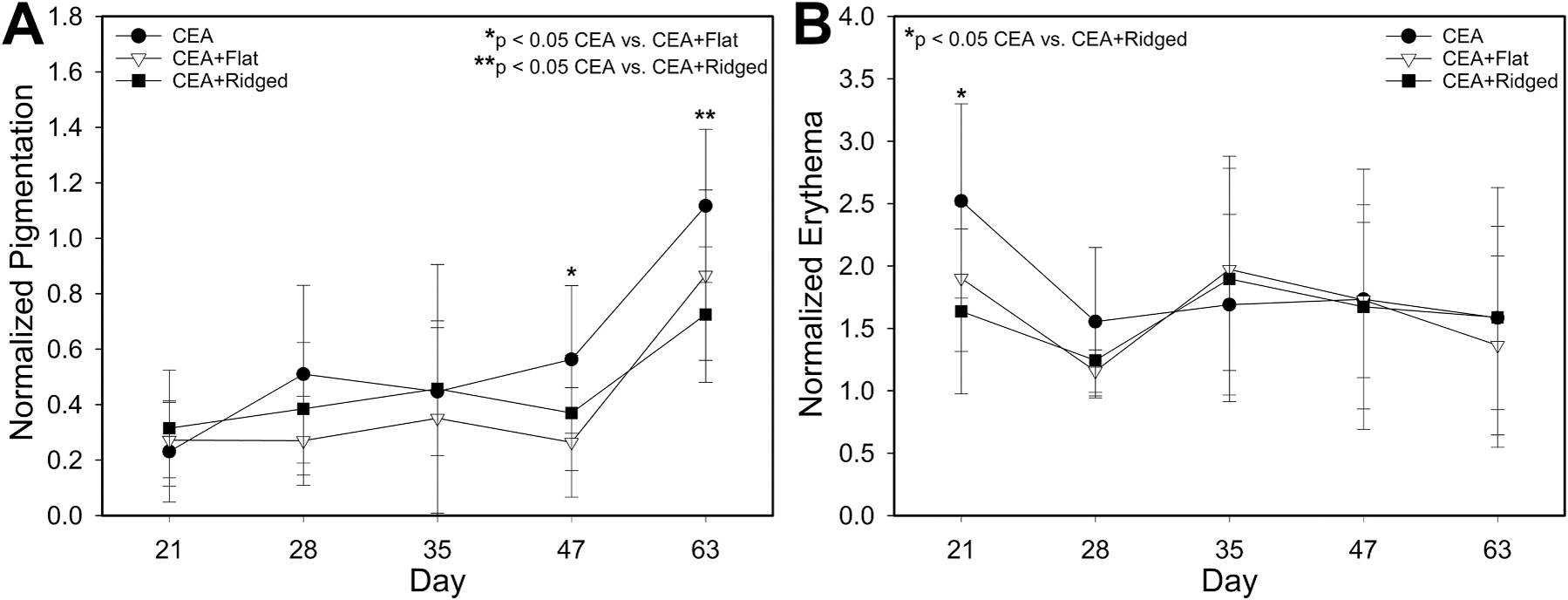
Quantitative analysis of pigmentation and erythema measurements of CEA, CEA+Flat and CEA+Ridged grafts at 21, 28, 35, 47 and 63 days post-grafting. A) Pigmentation was similar between groups through day 35. At days 47 and 63, average values for pigmentation were greater in the CEA group. B) Erythema was significantly decreased in CEA+Ridged grafts versus CEA grafts at day 21 after which erythema decreased in all groups.

No differences in engraftment rates were observed among treatment groups; however, improved tissue integration at the graft-wound boundary was observed in both the CEA+Flat and CEA+Ridged groups (Fig. 3). All grafts contracted in a similar manner over time, with CEA, CEA+Flat and CEA+Ridged grafts contracting to averages of 53.9, 59.0 and 54.3 % of their original size, respectively, over the 9-week experiment (Fig. A1). Pigmentation was similar between groups until day 35, after which pigmentation in CEA grafts increased more rapidly, significantly more than CEA+Flat grafts at day 47 and CEA+Ridged grafts at day 63 (Fig. 4A). In all groups, hyperpigmentation was observed at the graft border with a larger, hypopigmented interior (Fig. 3). On average, pigmentation of all graft conditions was statistically similar to that of normal pig skin at day 63. CEA grafts displayed increased erythema at early time points, through day 28, based on visual assessment (Fig. 3). Quantitative assessments of color showed that erythema was elevated in the CEA alone group compared to the CEA+Ridged grafts at day 21 (Fig. 4B).

**Fig. 5.**
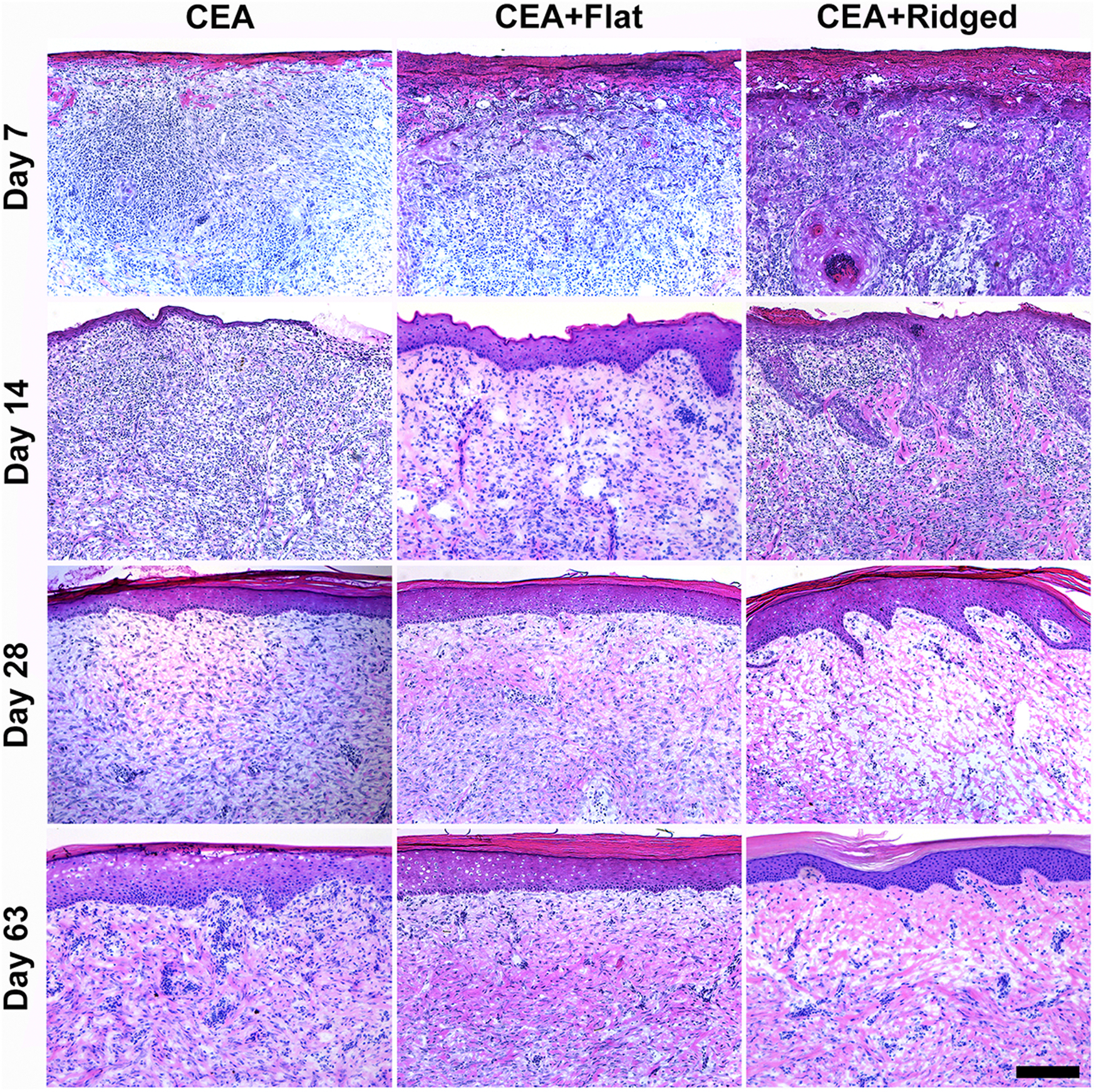
Representative images of H&E stained cross-sections of CEA, CEA+Flat and CEA+Ridged grafts at 7, 14, 28, and 63 days post-grafting. The presence of a dermal template, in CEA+Flat and CEA+Ridged grafts generally sped epidermal development and decreased variability, especially at early time points. Scale bar = 200 μm.

H&E stained cross-sections of CEA grafts showed that epidermal development was highly variable for the first 2 weeks after grafting (Fig. 5). At day 14, CEA grafts displayed a mix of well differentiated epidermis and regions of thin or discontinuous epidermis. CEA+Flat and CEA+Ridged grafts contained more uniform and continuous epidermal layers, even at day 7, and a thicker epidermis than that of CEA grafts at early time points. Rete ridges were observed in all CEA+Ridged grafts starting at day 14, and were the deepest at that time point. Sparse, shallow rete ridges were observed in some CEA+Flat grafts and only in one CEA graft at day 14. Shallow ridges were observed in more CEA+Flat and CEA grafts with time; however, these remained sparse.

**Fig. 6.**
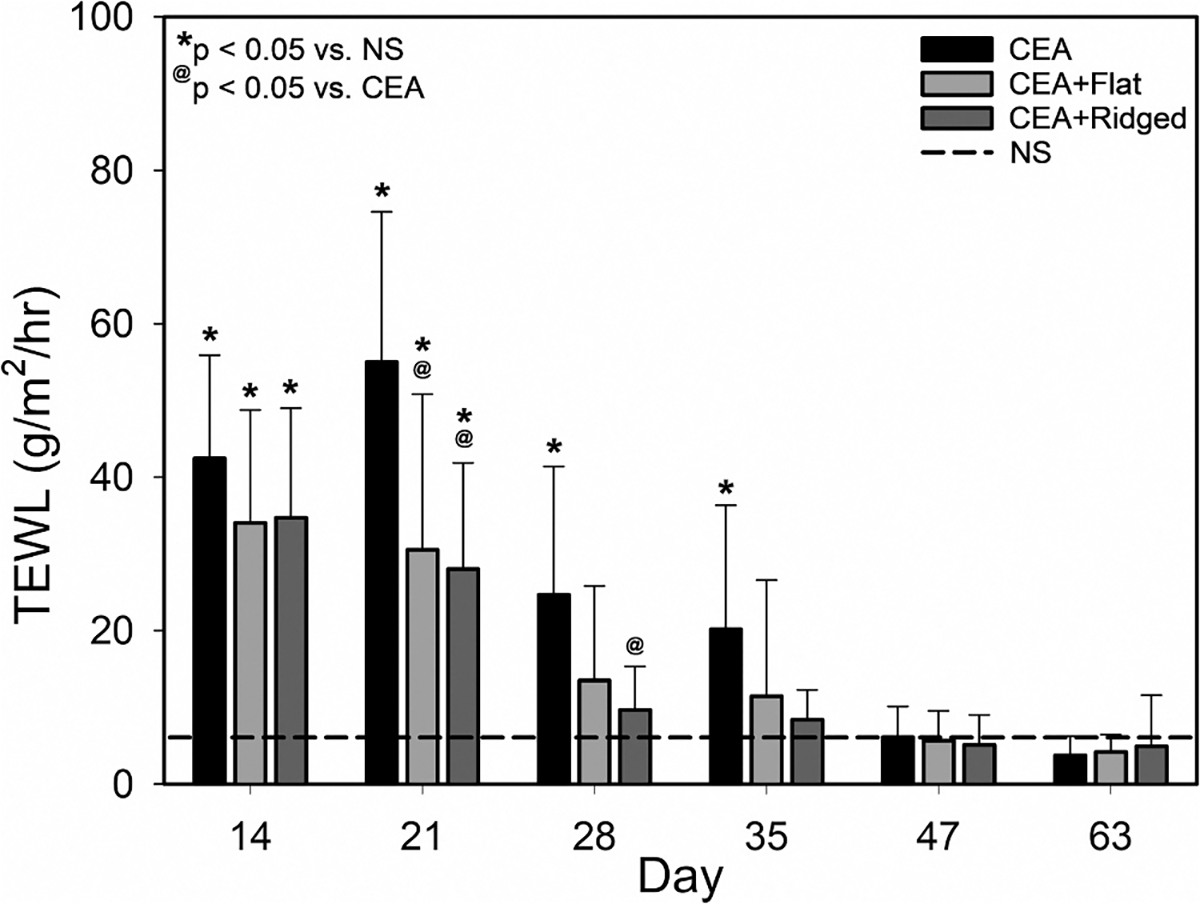
Transepidermal water loss measurements of CEA, CEA+Flat and CEA+Ridged grafts at 14, 21, 28, 35, 47 and 63 days post-grafting. Water loss through CEA+Flat and CEA+Ridged grafts was significantly decreased compared to the CEA group at day 21. By day 28, CEA+Flat and CEA+Ridged groups reached normal skin levels of transepidermal water loss whereas the CEA group required 47 days to reach normal skin levels. At days 21–35, the use of ridged dermal templates resulted in lower average transepidermal water loss and less variability.

Grafts were assessed for transepidermal water loss (TEWL) starting on day 14 post-grafting. TEWL from all graft conditions at days 14 and 21was significantly greater than that of normal pig skin (Fig. 6). TEWL for CEA grafts increased at day 21 and was significantly higher than that of CEA+Flat and CEA+Ridged grafts. At day 28, TEWL for CEA+Ridged grafts was significantly lower than TEWL for CEA grafts and by day 28, CEA+Flat and CEA+Ridged grafts were not significantly different than normal pig skin while CEA grafts remained elevated until day 35.

### 3.3. Graft biomechanics

By day 35, all grafts were sufficiently healed to prevent any possible damage from the suction required for biomechanical measurement with the BTC-2000^™^. Stiffness of all graft conditions were found to be statistically similar to normal pig skin at days 35, 47 and 63. Significant differences from normal pig skin were only observed for laxity and elasticity of CEA+Ridged grafts at day 47 (Fig. A2).

### 3.4. Immunohistochemical assessment

**Fig. 7.**
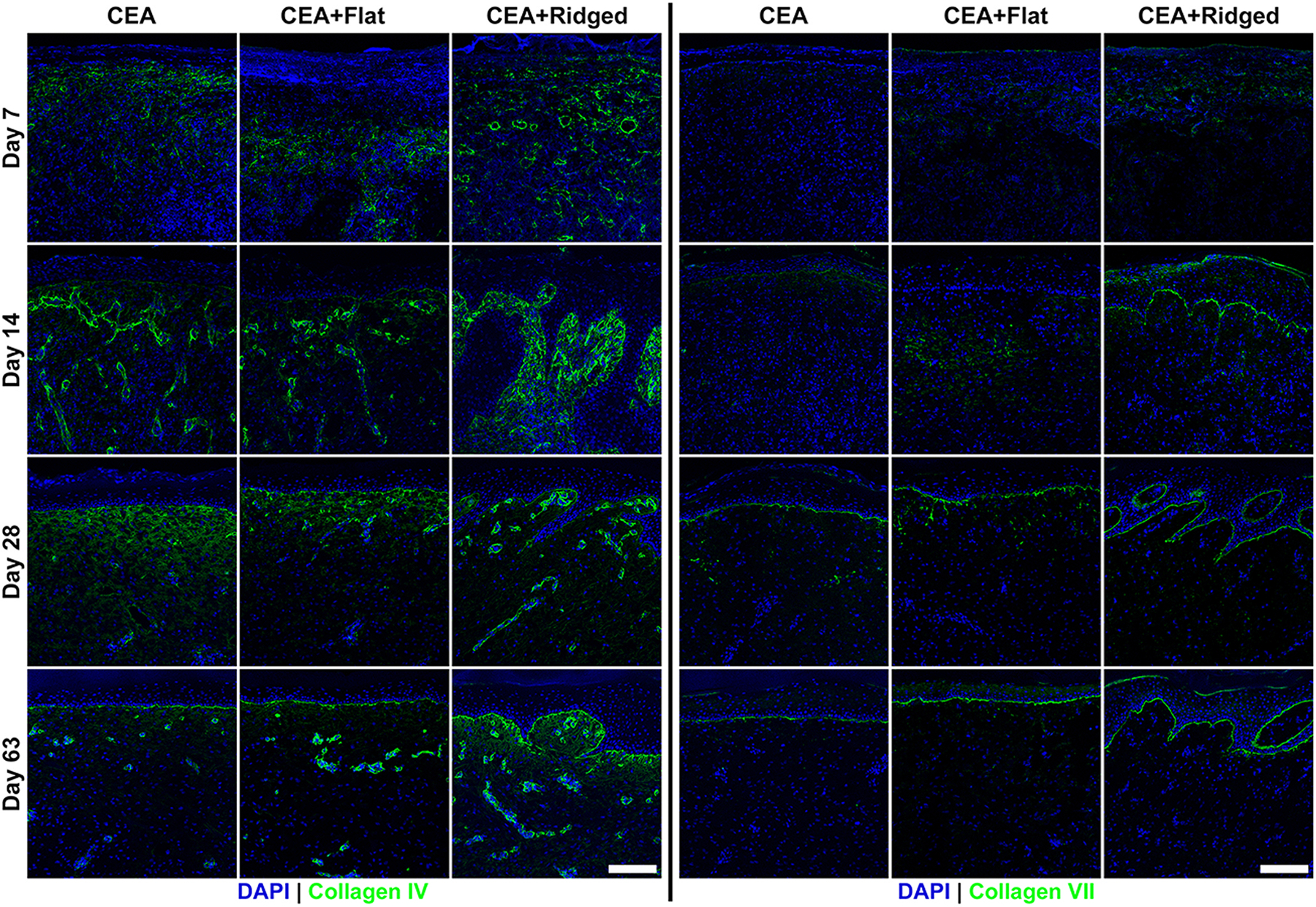
Immunohistochemical staining for collagen IV (green, left side) and collagen VII (green, right side) in CEA, CEA+Flat and CEA+Ridged grafts at 7, 14, 28 and 63 days post-grafting. Nuclei were counter-stained with DAPI (blue). Collagen IV was observed early (day 7) in the CEA+Ridged group with collagen IV at the basement membrane and in newly formed blood vessels. With time collagen IV staining remained the most intense and continuous at the DEJ in the CEA+Ridged group. Similarly, collagen VII was present at the DEJ in the CEA+Ridged group at day 14 whereas it is largely absent in the other groups at this time point. Sparse amounts of collagen VII were observed in the CEA group at days 28 and 63. Scale bar = 150 μm for all panels.

Basement membrane formation was assessed via collagen IV and collagen VII immunostaining. Collagen IV, which is present in the basement membrane of the dermal-epidermal junction (DEJ) and of blood vessels, was throughout the dermis of all graft conditions at day 7 post-grafting, both diffusely and more localized to developing blood vessels (Fig. 7). At day 14, collagen IV in CEA and CEA+Flat grafts appeared to be more localized to developing blood vessels, with very little positive staining at the DEJ. In contrast, CEA+Ridged grafts showed an increased concentration of collagen IV in the dermis just below the DEJ, in addition to collagen IV localization to basement membranes of blood vessels. Collagen IV became more localized to the DEJ and was slightly decreased overall at day 28. Increased positive collagen IV staining at the DEJ was observed at day 63, with the highest intensity seen in CEA+Ridged samples.

Deposition of collagen VII at the DEJ was more rapid and continuous in the CEA+Ridged grafts compared to the other groups (Fig. 7). Cells expressing collagen VII were observed in the dermis of the CEA+Flat and CEA+Ridged grafts, but not CEA only grafts, at day 7, and no collagen VII was localized to the DEJ in any group at this time point (Fig. 7). At day 14, small areas of diffuse collagen VII were observed in CEA and CEA+Flat grafts, while a continuous layer of collagen VII was present at the DEJ of CEA+Ridged grafts. By day 28, collagen VII increased at the DEJ in CEA and CEA+Flat grafts and was found across the length of the DEJ at day 63. Within the CEA+Ridged group, a thick layer of collagen VII was observed at the DEJ from day 14 through 63.

**Fig. 8.**
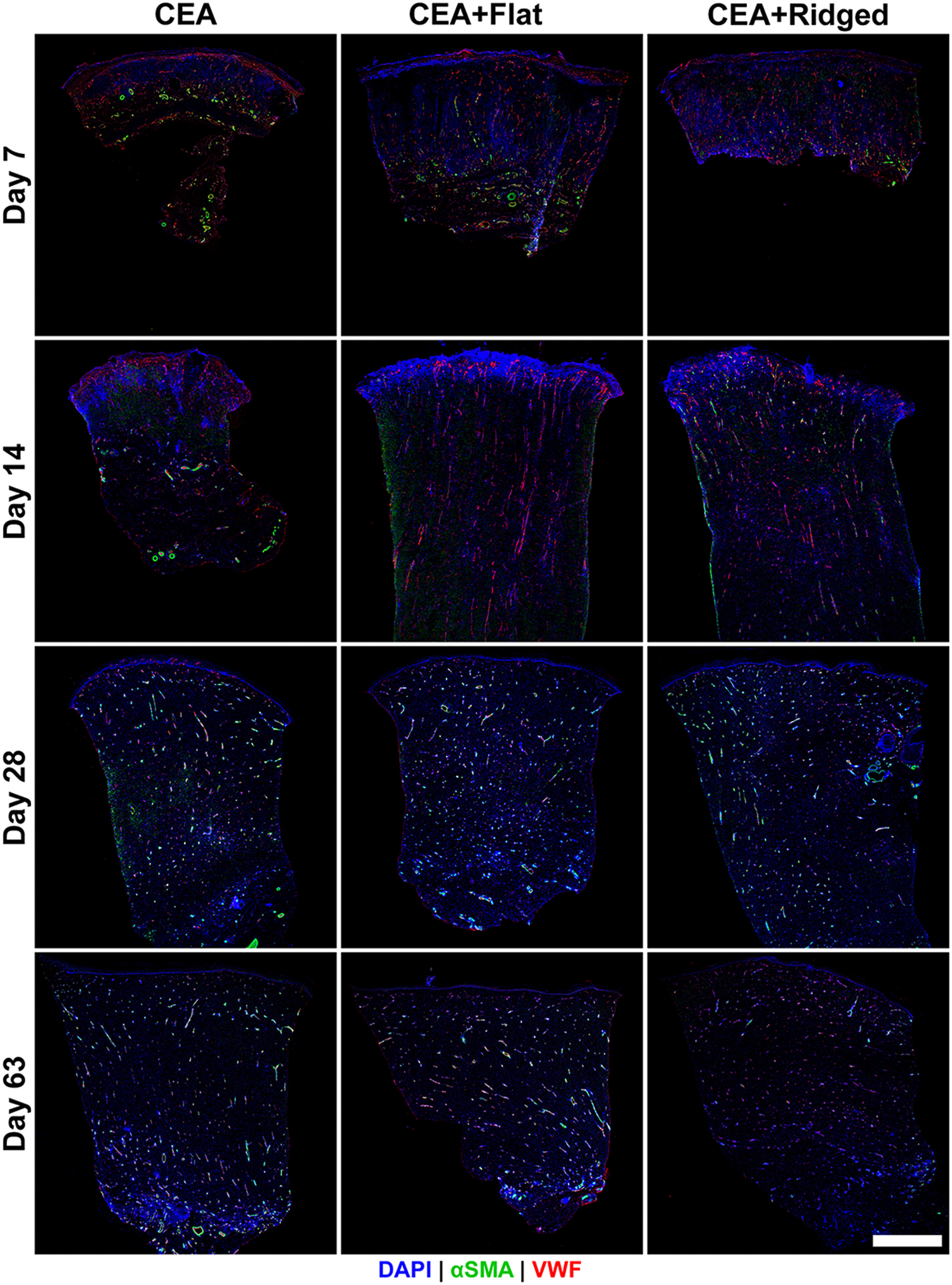
Immunohistochemical staining for alpha smooth muscle actin (green) and Von Willebrand Factor (red) in CEA, CEA+Flat and CEA+Ridged grafts at 7, 14, 28 and 63 days post-grafting. Nuclei were counter-stained with DAPI (blue). VWF was observed in all wound beds and throughout the dermal templates of CEA+Flat and CEA+Ridged grafts, with noticeable directionality at day 14 and a slight increase in vessel density in the CEA+Ridged group. Considerable remodeling occurred between days 14 and 28, with an increase in mature vessels found at day 28. Myofibroblast presence peaked at day 14 for all groups. Scale bar = 1.5 mm.

Blood vessel formation was assessed by immunostaining for VWF, which is produced by endothelial cells, and for α-SMA. Although α-SMA is expressed by myofibroblasts, in blood vessels it is expressed by pericytes; thus, the presence of α-SMA^+^ cells surrounding blood vessels indicates more mature vessels. At days 7 and 14 post-grafting, a large number of endothelial cells were observed in the wound bed of all grafts and in the dermal templates of CEA+Flat and CEA+Ridged grafts, with α-SMA being predominately localized to blood vessels at the bottom of the wound bed (Fig. 8). Interestingly, VWF staining of endothelial cells revealed numerous directionally oriented vessels running from the subcutaneous region to the epidermis in CEA+Flat and CEA+Ridged grafts. In all groups, there were fewer α-SMA-positive deep vessels and little to no myofibroblasts at day 7. At day 14, numerous myofibroblasts were observed in all groups; numbers of myofibroblasts decreased until day 63, with few myofibroblasts observed in any grafts by this time point. At days 28 and 63, directionality of VWF-positive vessels had largely subsided and colocalization of VWF and α-SMA was observed throughout the tissue in all groups. Quantification of VWF-positive staining in the dermis showed a trend for all groups of increased VWF presence at days 7 and 14 over days 28 and 63 (Fig. A3), indicating high angiogenic activity at early time points and resolution over time. Based on VWF staining, vessel density appeared highest in CEA+Ridged grafts at day 7, although no statistically significant differences were present among different groups or time points.

**Fig. 9.**
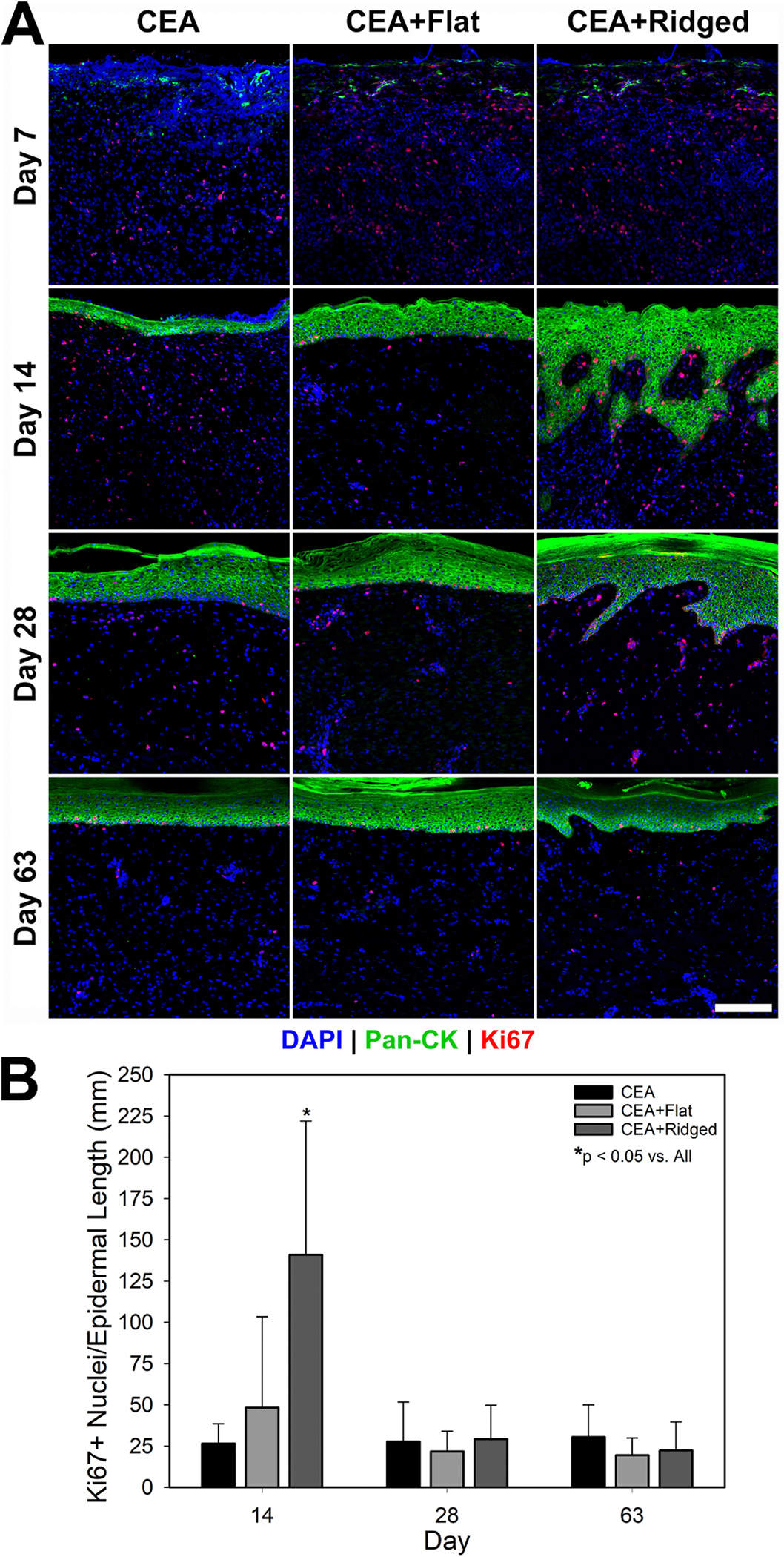
Immunohistochemical localization for proliferative keratinocytes in CEA, CEA+Flat and CEA+Ridged grafts at days 7, 14, 28 and 63 post-grafting. A) IHC staining for pan-cytokeratin (green) and Ki67 (red), with a counterstain for nuclei (DAPI; blue). At day 14, a large spike in number of Ki67^+^ nuclei was observed in the epidermis of CEA+Ridged grafts, as well as rete ridge development. Scale bar = 250 μm. B) Quantitative analysis of epidermal Ki67 positive nuclei normalized to the length of the epidermis. Epidermal Ki67^+^ nuclei were significantly increased in number in CEA+Ridged grafts at day 14, versus all groups and all time points.

Immunohistochemical staining for pan-cytokeratin and Ki67 was used to evaluate epidermal proliferation (Fig. 9). Ki67 ^+^ nuclei were seen throughout the CEA and dermal templates at day 7 post-grafting. As it was difficult to discern the boundary between the CEA and dermal template, and the CEA and wound bed, Ki67^+^ keratinocyte quantification was not performed at this day (Fig. 9A). On day 14, there appeared to be a much larger number of Ki67 ^+^ nuclei in the epidermis of CEA+Ridged grafts than the other groups, and quantitative analysis confirmed that there were significantly more Ki67 ^+^ nuclei per mm of epidermal length in CEA+Ridged grafts (Fig. 9B). This number significantly decreased on day 28, from which point on, all groups were similar.

## 4. Discussion

When used by experienced surgeons in burn centers with optimized care protocols, CEA can be an important adjunct for treatment of patients with large TBSA full-thickness burns. However, deficiencies attributable to lack of a dermal component have led users to call for development of more robust dermal substitutes to improve outcomes for patients treated with CEA [44]. For clinical application of CEA to excised burns, reported engraftment rates are extremely variable [7–11] and likely impacted by the skill and experience level of the surgeon and those responsible for dressing changes. In the current study, the combined use of the CEA and dermal template created a more robust graft, improving the ability to handle and surgically apply the graft. The surgeons performing the porcine grafting surgeries and subsequent dressing changes described here had extensive experience handling CEAs, resulting in the similarly high levels of engraftment observed for all conditions and CEA variability only being noticeable histologically.

A limitation of this study was that we were unable to non-invasively analyze graft biomechanics at early time points because the measurement, which involves negative pressure, could have damaged the grafts and confounded the analysis of engraftment. Thus, differences in biomechanics may have existed early post-grafting but were not able to be assessed due to this concern. Despite observed improvements in CEA+Flat and CEA+Ridged graft quality at the time of grafting and in the first two weeks post-grafting, these improvements did not translate into reduced wound contraction compared with wounds treated with only CEA. This could be due to the compliance of the scaffold, which may be unable to resist the contractile forces experienced in the wound bed and exerted by the transplanted fibroblasts. Sohutskay et al. showed that for collagen gels grafted onto full-thickness excisional wounds in rats, increases in the Young’s modulus of the scaffold corresponded with increased retention of the original wound area [45]. Therefore, a stiffer scaffold should be investigated in future studies to combat graft contraction.

Despite there being no observed differences in graft contraction, the addition of a dermal template resulted in more consistent epidermal formation at early time points. The markedly variable epidermal formation in CEA grafts observed in histological sections was likely due to areas of poor adhesion and loss of epidermal cell layers post-grafting, which are characteristic of CEA grafts clinically [2–5]. Dermal templates may have provided protection for the CEAs used in CEA+Flat and CEA+Ridged grafts, both during grafting as well as by retaining moisture within the wound bed post-grafting. The included fibroblasts likely also played an important role in epidermal development as fibroblasts and keratinocytes are known to interact in a double paracrine loop that regulates the wound healing process [46]. Fibroblast feeder layers have been observed to improve keratinocyte adhesion and proliferation *in vitro*, and the direct contact of the two cell types increases cytokine secretion over conditioned medium exposure, growth factor addition or indirect contact [47,48]. Here, at two weeks post-grafting, keratinocyte proliferation was improved in grafts that included a fibroblast-containing DT, CEA+Ridged grafts displayed more frequent and deeper ridges, and the number of Ki67 + keratinocytes in CEA+Ridged grafts was ~2.9 and ~5.3 fold higher than in CEA+Flat and CEA grafts, respectively. This is in agreement with previous studies performed using an immunodeficient mouse excisional wound model, where Ki67 + nuclei were significantly increased in laser micropatterned DTs over flat DTs when combined with CEAs [38], and when used to fabricate engineered skin grafts [29]. The ridged architecture would have provided increased surface area for the CEA to attach and to interact with fibroblasts. The micropattern ablations have a frequency of 841/cm^2^ and were previously quantified as having an average width of 78 ± 3 μm and an average depth of 539 ± 18 μm [29]. Clement et al. [26] found that keratinocytes in narrow channels of 50–100 μm were significantly more proliferative than in wider channels and flat substrates, and that $\beta_{1}^{\text{bri}}{\text{p63}}^{+}$ keratinocytes (putative epidermal stem cells) were more likely to cluster in channels than on flat substrates.

Though it was hypothesized that the presence of rete ridges would increase vascularization in the papillary dermis, no differences in erythema or blood vessel density, assessed via Mexameter or immunohistochemistry, were observed on days 28–63. The CEA alone group had significantly greater erythema on day 21; however, this is most likely due to proximity of the vessels to surface, as CEA group was placed directly onto the wound bed. Similarly, we anticipated enhanced pigmentation in the CEA+Ridged group due to increases in basement membrane length and crosstalk between the dermis and epidermis. In contrast, the CEA group was close to normal pigmentation by day 63 whereas both the CEA+Flat and CEA+Ridged groups remained slightly hypopigmented. Similarly, a prior study reported decreased rete ridges in dyschromic hypertrophic scars but no correlation between rete ridge number and pigmentation [49].

Early improvements in epidermal formation in DT-containing grafts translated into quicker restoration of epidermal barrier function to that of normal skin, which occurred by day 28, while this took almost 3 additional weeks for CEA-only grafts. This contrasts with our previous work in the immunodeficient mouse model, where CEAs grafted with laser micropatterned DTs took longer than those with flat DTs to decrease TEWL to that of normal mouse skin [38]. We attribute the more rapid epidermal barrier formation to recent improvements to dermal template fabrication implemented here, as well as differences in the animal models. The significantly improved strength and stiffness [50] of the lyophilized collagen used here, as opposed to the electrospun collagen used in prior mouse studies, was likely a factor in improved epidermal development. Importantly, the increased *in vitro* culture time of the DTs in the current study likely allowed for more fibroblast proliferation prior to laser micropatterning and grafting. Additionally, differences in the animal models, including more substantial dressing materials used to protect grafts in the pig model compared with those used in the mouse model [38], may have contributed to reduced post-grafting tissue damage, enabling more rapid epidermal maturation.

In this study CEAs were grafted with the dermal templates in a single stage procedure. Many prior studies have called for grafting dermal matrices to the wound bed for 10 or more days prior to CEA application [16,51–53]. For example, Soejima et al. applied Pelnac (Gunze International USA, Inc., New York, NY), a synthetic collagen-based wound dressing containing a silicone film layer, to full-thickness excisional wounds in male crossbreed swine for 10 days before the CEAs [52]. In a human clinical study, CEA were successfully used in conjunction with biodegradable temporizing matrix (BTM) to treat massive burn injuries (81 % TBSA). In this cohort, BTM was placed on the wound bed for 31–67 days prior to CEA application [16]. In these studies, the dermal matrices were acellular, thus time for cellular infiltration and vascularization was desired. In contrast, our dermal templates were pre-populated with allogeneic fibroblasts and vascularized quickly with small, well-formed vessels present by day 7. Future studies examining the optimal timing of CEA grafting post dermal template application, the role of micropatterned well size on CEA stability and epidermal development or studies to speed vascularization in the dermal template would be valuable to further improve outcomes with the CEAs.

## 5. Conclusions

Using fibroblast-seeded dermal templates as carriers for CEAs at the time of grafting improved handleability and reduced variability in early epidermal development. Epidermal barrier function was reestablished almost three weeks faster when CEA was combined with a dermal template, whether flat or ridged, compared with CEA alone. Further, the results suggest that inclusion of an avascular dermal template did not slow vascularization. Importantly, basement membrane formation occurred more rapidly in the presence of a dermal template, which is expected to reduce blistering post-grafting, a frequent complication of grafted CEA. Finally, grafting of CEA with a ridged dermal template resulted in more proliferative epidermal nuclei at early time points after grafting. Taken together, the results indicate that a dermal template used in conjunction with CEA can enhance CEA performance, providing an improved option for treatment of patients with very large full-thickness burn wounds.

## Supplementary Material

Supplementary Material

## Appendix A. Supporting information

Supplementary data associated with this article can be found in the online version at doi:10.1016/j.burns.2025.107613.

## Abbreviations

α-SMA: alpha smooth muscle actin
BTC: Biomechanical Tissue Characterization
BTM: biodegradable temporizing matrix
CEA: cultured epithelial autograft
DAPI: 4′,6-diamidino-2-phenylindole
DEJ: dermal-epidermal junction
DT: dermal template
FXCO_2_: fractional carbon dioxide
HBS: HEPES buffered saline
IACUC: Institutional Laboratory Animal Care and Use Committee
SD: standard deviation
STSG: split-thickness skin graft
TBSA: total body surface area
TEWL: transepidermal water loss
VWF: Von Willebrand Factor.
