# Supplementary material for: Laser micropatterned dermal templates support early rete ridge formation and basement membrane deposition when used with cultured epithelial autografts
Source: Burns. Author manuscript; Available in PMC 2026 Jul 4. (PMC13332489; doi:10.1016/j.burns.2025.107613)
Supplement: Supplementary Material [file NIHMS2176871-supplement-Supplementary_Material.pdf]

Appendix: Supplementary Material

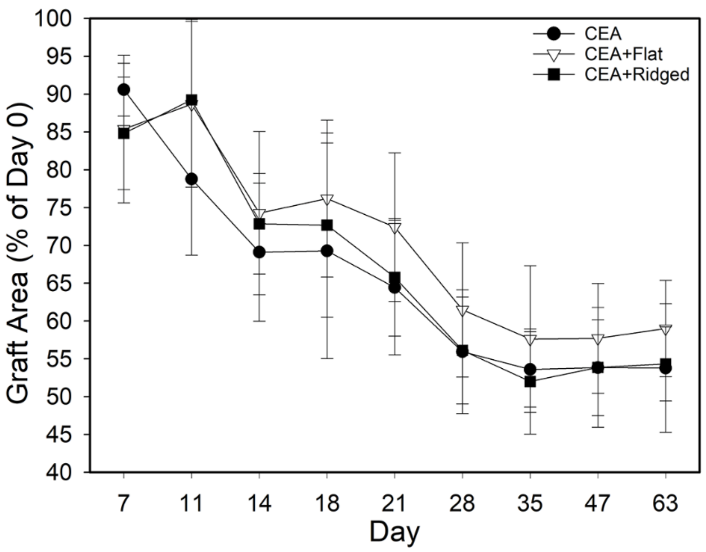

Suppl. Fig. 1. Area of CEA, CEA+Flat and CEA+Ridged grafts normalized to graft size at the time of grafting. Graft contraction was similar for all graft conditions at days 7, 11, 14, 18, 21, 28, 35, 47 and 63 post-grafting.

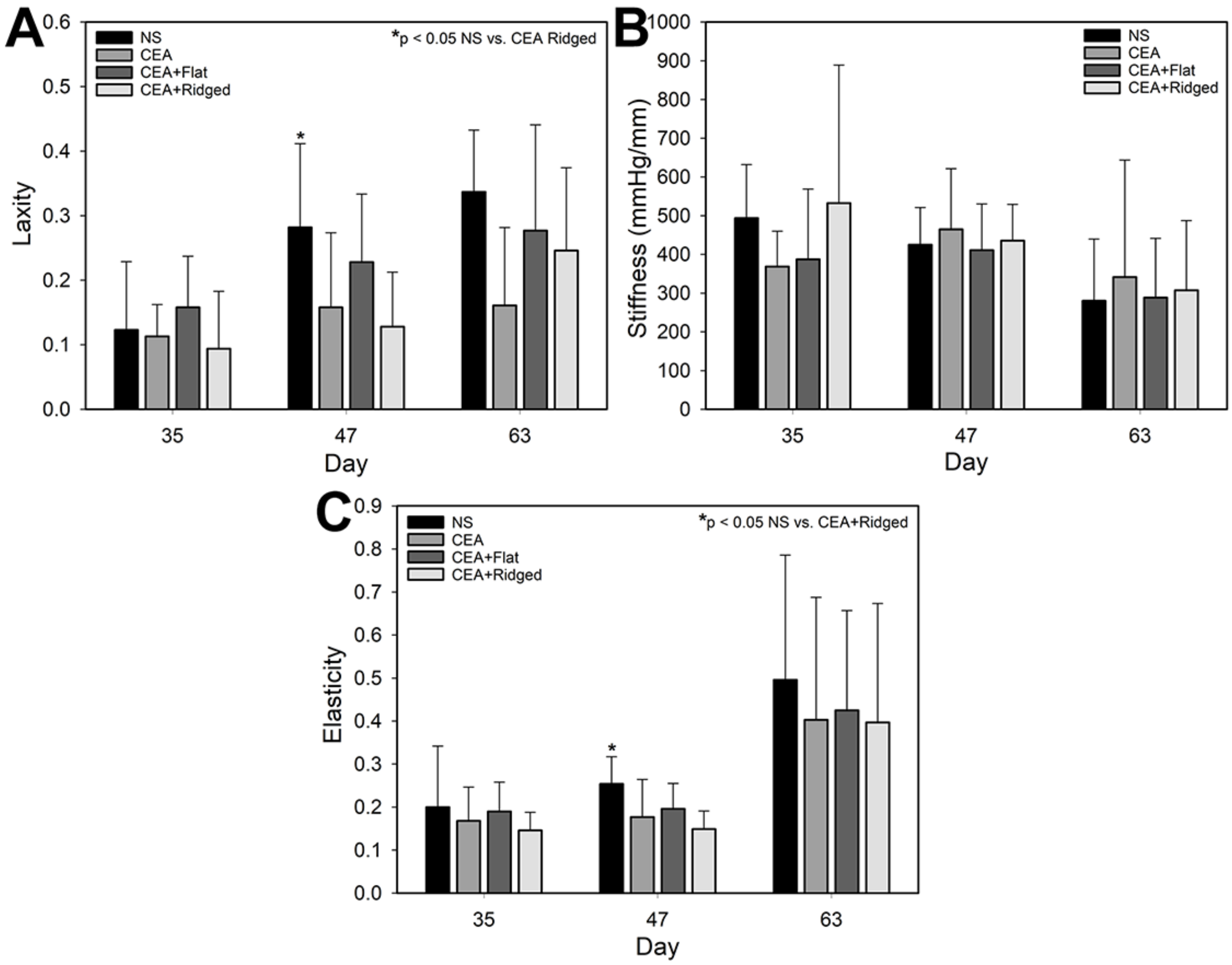

Suppl. Fig. 2. Biomechanical analysis of normal pig skin (NS) and CEA, CEA+Flat and CEA+Ridged grafts at 35, 47 and 63 days post-grafting. Laxity and elasticity were decreased in CEA+Ridged grafts from NS at day 47, though no statistical difference was observed between graft conditions at all time points.

Appendix: Supplementary Material

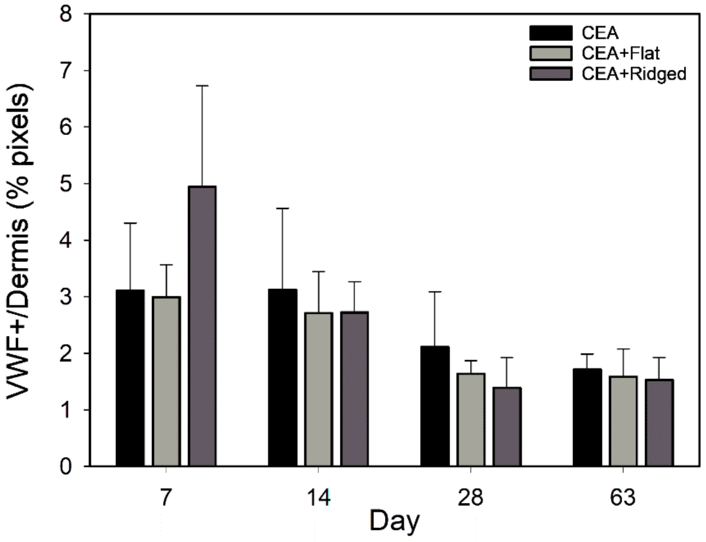

Suppl. Fig. 3. Quantitative analysis, reported average ± SD, of Von Willebrand Factor (VWF) positive pixels as a percentage of the total pixels of the dermis in CEA, CEA+Flat and CEA+Ridged grafts at 7, 14, 28 and 63 days post-grafting. There was no statistical difference between groups ($p > 0.05$), despite the avascular dermal templates of CEA+Flat and CEA+Ridged grafts, which displayed significantly increased VWF presence at day 7 over days 28 and 63.
